# Oncostatin M is a novel biomarker for coronary artery disease – A possibility as a screening tool of silent myocardial ischemia for diabetes mellitus

**DOI:** 10.1016/j.ijcha.2021.100829

**Published:** 2021-06-26

**Authors:** Shohei Ikeda, Koichi Sato, Morihiko Takeda, Keita Miki, Kentaro Aizawa, Tsuyoshi Takada, Koji Fukuda, Nobuyuki Shiba

**Affiliations:** Department of Cardiovascular Medicine, International University of Health and Welfare Hospital, Tochigi, Japan

**Keywords:** OSM, Coronary artery diseases, Silent myocardial ischemia, Biomarker, Diabetes mellitus, BNP, brain natriuretic peptide, BMI, body mass index, CACS, coronary computed tomography calcium score, CAD, coronary artery disease, CAG, coronary angiography, DM, diabetes mellitus, EF, ejection fraction, FFR, fractional flow reserve, HDL-C, high-density lipoprotein-cholesterol, HF, heart failure, iFR, instantaneous wave-free ratio, LDL-C, low-density lipoprotein-cholesterol, LVEF, left ventricular ejection fraction, OSM, oncostatin M, PCI, percutaneous coronary intervention, YAP, yes-associated protein

## Abstract

**Objective:**

Oncostatin M (OSM) is an inflammatory cytokine of the interleukin-6 family which plays a crucial role in the pathogenesis of atherosclerosis. Therefore, we tested our hypothesis that serum OSM levels are increased in patients with coronary artery diseases (CAD).

**Methods and results:**

Serum OSM level was measured by sandwich technique immunoassay in 315 consecutive patients and who underwent coronary angiography at the International University of Health and Welfare Hospital from April 2019 to March 2021. A diagnosis of CAD was made in 169 patients. Serum OSM levels were significantly higher in patients with significant coronary stenosis compared to those without it. [123.0 ± 46.7 pg/mL (n = 169) vs. 98.3 ± 47.9 pg/mL (n = 146), p < 0.001]. A positive correlation was noted between serum OSM levels and severity and complexity of coronary stenosis. Importantly, the coronary revascularization significantly decreased the serum OSM levels. We furthermore detected a positive correlation between serum OSM levels and HbA1c levels. Finally, our data suggested that 120 pg/mL of serum OSM was the potential cutoff value for screening of silent myocardial ischemia related with diabetic mellitus (DM).

**Conclusion:**

Serum OSM can be a novel biomarker for CAD and may be useful for the screening of asymptomatic CAD in patients with DM.

## 1 Introduction

Coronary artery disease (CAD) is the most common type of heart disease and is a major complication of diabetes mellitus (DM) [1], [2]. The complicated lesions, such as diffuse and calcified lesion, are often presented in the coronary stenosis related with DM [3]. The relationship between complicated nonobstructive and atherosclerotic calcification lesions with DM-related silent myocardial ischemia has been reported, but its mechanism remains unsolved [4], [5]. The diagnostic delay often causes fatal cardiovascular events, and then it is desired to develop the specific screening tool for silent myocardial ischemia. Several studies have investigated the effective screening method for patients with DM and CAD that exhibited silent myocardial ischemia including the adenosine–stress radionuclide myocardial perfusion imaging or coronary computed tomography angiography [6], [7], however, so far, there had been no appropriate method. On the other hand, several biomarkers were investigated and proposed for CAD , such as hsCRP (high sensitivity C-reactive protein), brain natriuretic peptide (BNP) and adipsin, but they were not enough for CAD related with DM [8], [9], [10].

Oncostatin M (OSM) is an inflammatory cytokine that belongs to the interleukin-6 family and is involved in various cardiovascular diseases [11], [12], [13]. OSM contributes to destabilization of atherosclerosis and the development of vessel calcification [14], [15]. We recently demonstrated that Yes associated protein (YAP) -OSM pathway played an important role in cardiac dedifferentiation which causes cardiac dysfunction [16]. We furthermore reported that the YAP-OSM pathway was involved in the diabetic cardiomyopathy [17]. Therefore, we hypothesized that YAP-OSM pathway plays a crucial role in CAD, especially CAD related with DM, and that the measurement of its activity is a potential biomarker.

## 2 Methods

### 2.1 Study patients

Consecutive patients who underwent elective coronary angiography (CAG) at the International University of Health and Welfare Hospital in Japan from April 2019 to March 2021 were included in this study. CAG was performed upon the discretion of cardiologists, based on the presence of chest symptoms and signs of CAD or for follow-up after previous percutaneous coronary intervention (PCI). For cases that underwent CAG more than once, only the data obtained at the time of the first CAG were included our analysis. Patients with unstable angina or acute and old myocardial infarction; cardiac dysfunction; valvular or congenital heart disease; chronic kidney disease, including those on dialysis; malignant tumor; or inflammatory disease, such as collagen disease, and preoperative patients were excluded in this study. For this study, coronary stenosis was evaluated by 2 or more cardiologists. CAD was defined by narrowing of the coronary lumen by >90% or by performing fractional flow reserve (FFR)/ instantaneous wave-free ratio (iFR). The adaptation of the revascularization was finally judged by 2 or more cardiologist.

### 2.2 Study approval

The ethics committee of the International University of Health and Welfare Hospital approved the study protocol, and all patients provided written informed consent. The authors had full access to and take full responsibility for the integrity of the data.

### 2.3 Measurement of serum levels of OSM

Blood samples were collected and centrifuged for 10 min at 2500 g within 30 min of blood collection, and aliquots were stored at − 80 °C. Serum levels of OSM were measured by sandwich technique immunoassay using human OSM ELISA kit (Lot: ab215543, Abcam, USA), according to the manufacturer’s instructions. To investigate effect with the revascularization, we checked serum OSM levels before and after PCI in the CAD patients with 1-vessel stenosis (n = 115).

### 2.4 Measurement of coronary computed tomography calcium score (CACS)

Coronary computed tomography angiography with 320-row detector (Aquilion ONE, Toshiba Medical Systems, Otawara, Japan) was used. To measure coronary CT calcium score (CACS), ZIOSTATION (M900, Ziosoft Inc., Tokyo, Japan) was used. A calcified lesion was defined as >3 contiguous pixels with a peak attenuation of at least 130 Hounsfield Units (HU). CACS of the all patients who was underwent coronary CT scan before CAG (n = 99) were measured by 2 or more radiographers in a blind manner.

### 2.5 Data collection

The medical records were used to collect baseline demographic data, including age, sex, body mass index (BMI), medication, and plasma brain natriuretic peptide. Left ventricular ejection fraction (LVEF) was measured on echocardiography. The cardiovascular risk was assessed in terms of the presence of DM, hypertension, dyslipidemia, smoking, aging, and obesity. Patients with hypertension were assessed as being at risk if their blood pressure was ≥140/90 mmHg or if they had a history of antihypertensive drug use. Patients with DM were defined as those who were previously diagnosed as DM or treated with hypoglycemic drug or insulin. Patients with dyslipidemia were assessed as being at risk if their low-density lipoprotein cholesterol was ≥140 mg/dL or high-density lipoprotein cholesterol was ≤40 mg/dL or if they were taking a lipid lowering drug. Patients with obesity were assessed as being at risk if their BMI was ≥25 kg/m^2^. Several cardiologists classified the lesions on CAG, based on the ACC/ AHA Task Force stenosis characteristic classification.

### 2.6 Statistical analysis

Results were expressed as mean ± standard error of mean. Continuous variables were presented as median and interquartile range, and categorical variables were presented as number and percentage. Baseline characteristics were compared among quartiles using the chi square test for categorical variables and the Wilcoxon or Kruskal–Wallis rank-sum test for continuous variables, as appropriate. The optimal cutoff value was determined by ROC curve analysis, as previously described [8]. Student’s *t*-test was used for comparisons between two groups, and Dunnett’s multiple comparison of means was used for multi-group comparison after analysis of variance. Univariate and multivariate Cox proportional hazard models were applied to determine hazard ratio and 95% confidence interval. Analyses were performed with SPSS (SPSS, Chicago, IL, USA). A p value of <0.05 was considered statistically significant.

## 3 Results

### 3.1 Clinical characteristics

The baseline clinical characteristics are shown in Table 1. The average age of all patients was 72.6 ± 8.9 years, and 78% were men. All patients had normal cardiac function and had an average LVEF of 62.3 ± 6.6%. There were 169 patients with CAD in this study. In addition, there were 144 patients with DM (46%) in this study. In patients with DM, the average HbA1c was 6.48 ± 0.9%, and the mean duration of diabetes was 5.42 ± 2.8 years. CAD was diagnosed in 83 patients with DM; of these, 72 exhibited no typical chest symptoms of chest pain or dyspnea and were considered to have silent myocardial ischemia. We furthermore paid attention to sex-difference (Online Table 1). In this study, the mean age of male was significantly younger than that of female. There was not the significant difference in the other points.

**Table 1:** Patient Characteristics - presence or absence of CAD-

	CAD positive	CAD negative	Total	P value
Number	169	146	315	
Male (%)	127 (75)	119 (82)	246 (78)	0.219
Age, years	72.2 ± 9.0	73.1 ± 8.8	72.6 ± 8.9	0.346
BMI, kg/m^2^	23.7 ± 2.9	23.4 ± 2.5	23.6 ± 2.7	0.310
Systolic BP, mmHg	136.5 ± 10.0	138.4 ± 10.2	137.4 ± 10.1	0.087
Diastolic BP, mmHg	78.1 ± 7.2	79.2 ± 5.9	78.6 ± 6.6	0.081
Heart rate, bpm	73.0 ± 4.8	73.7 ± 5.6	73.3 ± 5.2	0.272
eGFR, mL/min/ 1.73 m^2^	61.1 ± 5.6	61.9 ± 4.4	61.5 ± 5.2	0.194
BNP, pg/ml	13.2 ± 8.1	13.6 ± 8.6	13.4 ± 8.3	0.653
HbA1c, %	6.61 ± 1.0	6.33 ± 0.8	6.48 ± 0.9	0.007
LVEF, %	62.1 ± 6.3	62.6 ± 6.9	62.3 ± 6.6	0.541
hs-CRP, mg/dl	0.30 ± 0.36	0.32 ± 0.46	0.31 ± 0.41	0.632
LDL-C, mg/dl	96.4 ± 32.0	95.6 ± 30.6	96.0 ± 31.2	0.808
HDL-C, mg/dl	49.4 ± 12.9	47.8 ± 11.5	48.7 ± 12.3	0.243
OSM, pg/ml	123.0 ± 46.7	98.3 ± 47.9	111.5 ± 48.8	<0.001

Medical history				
DM (%)	83 (49)	61 (42)	144 (46)	0.193
Hypertension (%)	140 (83)	129 (88)	269 (85)	0.201
Dyslipidemia (%)	134 (79)	114 (78)	248 (79)	0.890
Smoking (%)	90 (53)	65 (45)	155 (49)	0.140

Medication				
Aspirin (%)	143 (85)	88 (60)	231 (73)	<0.001
Clopidogrel (%)	53 (31)	35 (24)	88 (28)	0.166
Prasugrel (%)	31 (18)	6 (4)	37 (12)	<0.001
ACEI/ ARB (%)	108 (64)	106 (73)	214 (68)	0.099
β-blocker (%)	108 (64)	51 (35)	159 (51)	<0.001
CCB (%)	106 (63)	83 (57)	189 (60)	0.301
Statin (%)	109 (64)	93 (64)	202 (64)	0.883
Insulin (%)	28 (17)	9 (6)	37 (12)	0.005
DPP4 inhibitor (%)	64 (38)	41 (28)	105 (33)	0.073
SGLT2 inhibitor (%)	16 (10)	5 (3)	21 (7)	0.041

### 3.2 Serum OSM level in patients with CAD

**Fig. 1:**
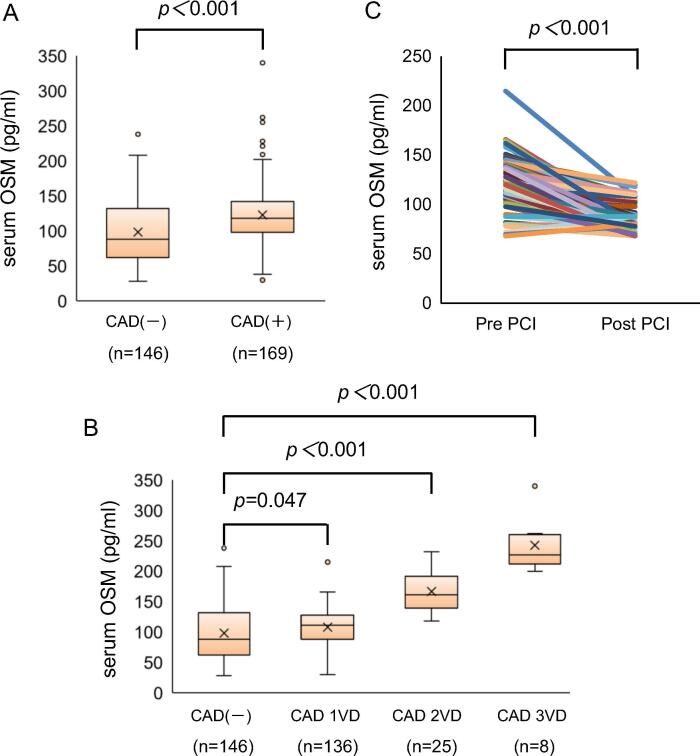
Serum OSM level in patients with CAD. (A) Serum OSM level in patients with CAD and without CAD (B) Serum OSM levels in patients with 1-, 2-, or 3-vessel disease (VD). (C) The change of serum OSM levels in patients with CAD-1VD before and after percutaneous coronary intervention (PCI).

Serum OSM level was significantly higher in patients with coronary organic stenosis compared with those without stenosis [123.0 ± 46.7 pg/mL (n = 169) vs. 98.3 ± 47.9 pg/mL (n = 146), p < 0.001] (Fig. 1A and Table 1). Moreover, serum OSM level increased with the severity of angiographic CAD (Fig. 1B). ROC curve analysis showed the area under the ROC curve was 0.65 (Online Fig. 1). Importantly, compared with the phase before PCI, serum OSM level was significantly decreased after PCI among 115 CAD patients with 1-vessel stenosis (Fig. 1C). We furthermore

found that serum OSM level was most relevant factor with CAD in this study (Online Table 2).

### 3.3 Correlation between serum OSM and obesity

**Fig. 2:**
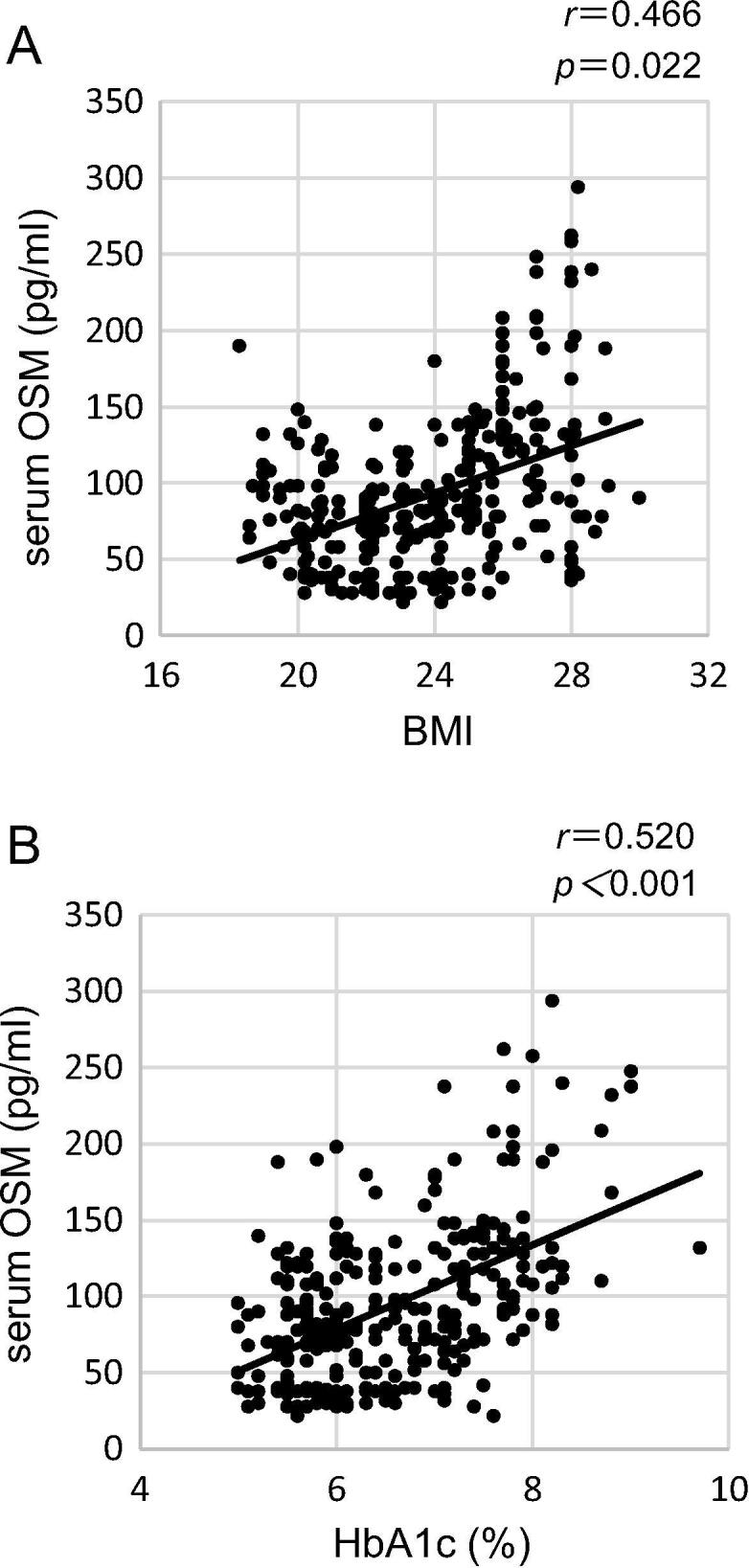
Correlation between serum OSM and obesity. Serum OSM had positive correlations with BMI (A) and HbA1c (B).

Next, we were interested in the source of OSM. Sanchez-Infantes D et al previously reported that OSM is produced in adipose tissue and is regulated in conditions of obesity and DM [18]. Therefore, we investigated the association between OSM and BMI. As shown in Fig. 2A and 2B, serum OSM had positive correlations with BMI and HbA1c. Based on our results on the positive correlations of serum OSM with BMI and HbA1c, we thought that OSM was very likely to be derived from adipose tissue.

### 3.4 Serum OSM level in patients with DM and CAD

**Fig. 3:**
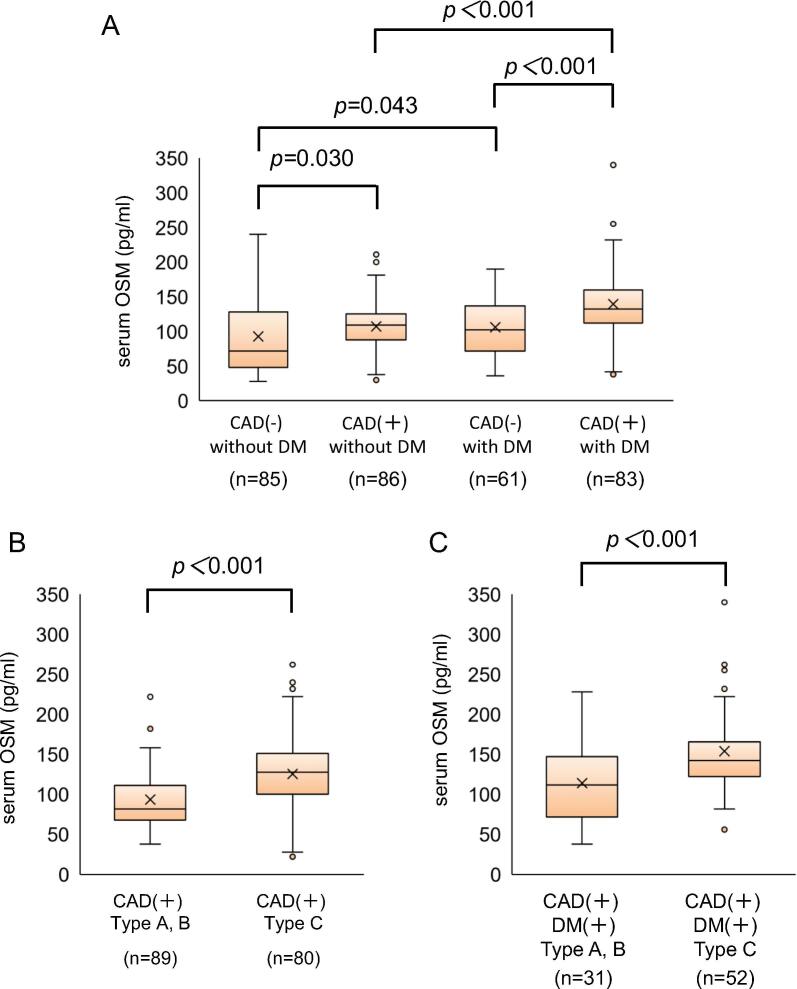
Serum OSM level in patients with DM and CAD. (A) Among patients with CAD, serum OSM level was significantly high in patients with DM. (B) Among patients with CAD, serum OSM level was significantly high in those who had the Type C AHA/ACC Task Force stenosis characteristic classification. (C) Among patients with DM and CAD, serum OSM level was significantly high in those who had the Type C AHA/ACC Task Force stenosis characteristic classification.

Interestingly, serum OSM level was higher in patients with DM than in those without DM [125.2 ± 50.3 pg/mL (n = 144) vs. 99.9 ± 44.4 pg/mL (n = 171), p < 0.001] (Table 2). Notably, among patients with DM, serum OSM level was significantly higher in those with CAD than in those without CAD [139.2 ± 52.8 pg/mL (n = 83) vs. 106.1 ± 39.7 pg/mL (n = 61), p < 0.001] (Fig. 3A). Furthermore, among patients with CAD, serum OSM level was significantly higher in those DM than in those without DM. [139.2 ± 52.8 pg/mL (n = 83) vs. 107.3 ± 33.2 pg/mL (n = 86), p < 0.001] (Fig. 3A) (see Table 3).

**Table 2:** Patient Characteristics - presence or absence of DM -

	DM positive	DM negative	Total	P value
Number	144	171	315	
Male (%)	114 (79)	132 (77)	246 (78)	0.673
Age, years	73.3 ± 8.6	72.1 ± 9.2	72.6 ± 8.9	0.204
BMI, kg/m^2^	23.7 ± 2.9	23.5 ± 2.5	23.6 ± 2.7	0.478
BNP, pg/ml	14.4 ± 9.0	12.6 ± 7.7	13.4 ± 8.3	0.063
HbA1c, %	7.33 ± 0.7	5.76 ± 0.3	6.48 ± 0.9	<0.001
LVEF, %	62.1 ± 7.1	62.4 ± 6.2	62.3 ± 6.6	0.693
hs-CRP, mg/dl	0.30 ± 0.40	0.31 ± 0.42	0.31 ± 0.41	0.746
LDL-C, mg/dl	95.2 ± 33.0	96.7 ± 29.8	96.0 ± 31.2	0.670
HDL-C, mg/dl	48.0 ± 12.5	49.2 ± 12.1	48.7 ± 12.3	0.366
OSM, pg/ml	125.2 ± 50.3	99.9 ± 44.4	111.5 ± 48.8	<0.001

Medical history				
CAD (%)	83 (58)	86 (50)	169 (54)	0.193
Hypertension (%)	120 (83)	149 (87)	269 (85)	0.341
Dyslipidemia (%)	115 (80)	133 (78)	248 (79)	0.653
Smoking (%)	71 (49)	84 (49)	155 (49)	0.974
Obesity (%)	55 (38)	47 (28)	102 (32)	0.043
Chest symptom (%)	28 (19)	66 (39)	94 (30)	<0.001

Medication				
Aspirin (%)	102 (71)	129 (75)	231 (73)	0.357
Clopidogrel (%)	45 (31)	43 (25)	88 (28)	0.229
Prasugrel (%)	20 (14)	17 (10)	37 (12)	0.278
ACEI/ ARB (%)	92 (64)	122 (71)	214 (68)	0.158
β-blocker (%)	79 (55)	80 (47)	159 (51)	0.153
CCB (%)	84 (58)	105 (61)	189 (60)	0.580
Statin (%)	100 (69)	102 (60)	202 (64)	0.071
Insulin (%)	37 (26)	0 (0)	37 (12)	<0.001
DPP4 inhibitor (%)	105 (73)	0 (0)	105 (33)	<0.001
SGLT2 inhibitor (%)	21 (15)	0 (0)	21 (7)	<0.001

**Table 3:** Factors associated with CAD among DM patients (Logistic regression analysis).

	Univariable analysis			Multivariable analysis		
	OR	95 %CI	*P*-value	OR	95 %CI	*P*-value
Age	0.978	0.941–1.017	0.269			
Gender	1.000	0.447–2.235	1.000			
HT	0.438	0.174–1.099	0.079	0.381	0.141–1.025	0.056
DL	0.771	0.340–1.748	0.534			
Smoking	1.653	0.856–3.194	0.135	2.300	1.108–4.775	0.025
BMI	0.997	0.891–1.115	0.957			
BNP	0.981	0.946–1.018	0.315			
HbA1c	1.884	1.103–3.219	0.020			
LVEF	0.986	0.942–1.033	0.565			
hs CRP	0.888	0.387–2.036	0.779			
LDL	0.992	0.982–1.002	0.119	0.989	0.978–1.000	0.052
OSM120	2.471	1.264–4.831	0.008	2.958	1.431–6.115	0.003

### 3.5 Serum OSM level in patients with DM and CAD that had complicated coronary artery lesions

Based on previous reports that complicated nonobstructive and atherosclerotic calcified lesions in patients with DM were related with silent myocardial ischemia [4], [5], we next paid attention to the presence or absence of the specific complicated lesions, such as diffuse and calcified lesions, which belong to the Type C AHA/ACC Task Force stenosis characteristic classification. Among patients with CAD, serum OSM level was significantly higher in those who had Type C lesions than in those who had Type A or B lesions [139.7 ± 51.4 pg/mL (n = 80) vs. 108.8 ± 36.8 pg/mL (n = 89), p < 0.001] (Fig. 3B). Furthermore, among patients with CAD and DM, serum OSM level was especially higher in those who had Type C lesions than in those who had Type A or B lesions [154.2 ± 50.4 pg/mL (n = 52) vs. 114.2 ± 47.8 pg/mL (n = 31), p < 0.001] (Fig. 3C). In fact, among CAD patients, multivariate analysis with stepwise selection could revealed that most relevant factors with CAD with Type C lesions were the presence of DM and HbA1c level (Online Table 3). We furthermore investigated CACS in the patients who underwent coronary CT scan before CAG (n = 94). Importantly, we revealed the significant correlation between serum OSM level and CACS (Online Fig. 2). In addition, we identified that, among patients with CAD who exhibited silent myocardial ischemia, serum OSM level was significantly higher in those with DM than in those without DM. [140.1 ± 47.5 pg/mL (n = 72) vs. 105.4 ± 26.2 pg/mL (n = 45)), p = 0.042] (Online Fig. 3).

### 3.6 Optimal cutoff value for serum OSM for CAD screening in patients with DM

Our data revealed that serum OSM level was most relevant factor with CAD (Online Table 2). In addition, our data suggested that serum OSM level is a potential biomarker of DM-related myocardial ischemia. For CAD screening, we tried to determine the optimal cutoff value for serum OSM in patients with DM. On ROC curve analysis, we determined 120 pg/mL as the optimal cutoff value for serum OSM as a CAD screening tool in patients with DM. There were significant differences in age; HbA1c; and number of cases of asymptomatic CAD dyslipidemia between patients with serum OSM level ≥ 120 pg/mL and those with serum OSM level < 120 pg/mL. Furthermore, among patients with DM, multivariate analysis with stepwise selection from the logistic model using all candidate covariates revealed smoking and serum OSM ≥ 120 pg/mL as the predictors of silent myocardial ischemia (Table 2).

## 4 Discussion

In this study, we investigated the utility of serum OSM level as a biomarker for the diagnosis of CAD. Our results showed that serum OSM level was relatively high in patients with CAD. Furthermore, a positive correlation was noted between serum OSM levels and severity and complexity of coronary stenosis. Notably, we furthermore showed that serum OSM level was relatively high in patients with DM and CAD, especially in those who exhibited silent myocardial ischemia. Finally, our data suggested that 120 pg/mL of serum OSM was the potential cutoff value for screening of silent myocardial ischemia related with DM.

In a recent *in vitro* study that used human vascular smooth muscle cells, OSM derived from plaque macrophages was found to contribute to the development of atherosclerotic calcification [15]. Interestingly, another recent research demonstrated the involvement of osteal tissue macrophages in endplate osteosclerosis through the OSM–YAP signaling axis [19]. Consistently, our results indicated that increased serum OSM level was related with CAD, especially with complicated lesions. In addition, our data revealed the significant correlation between serum OSM level and CACS. In fact, CAD patients who had 2 or 3 significant stenosis exhibits especially high CACS, whereas CAD patients who had 1-vessel stenosis did not show significantly high CACS compared with the patients without CAD. Notably, OSM was previously reported to be produced in adipose tissue and is regulated in conditions of DM and obesity [18]. In this present study, we found that serum OSM level correlated with DM and obesity. Furthermore, we previously demonstrated that adipsin, which is a member of the trypsin family of peptidases and is mainly secreted from adipocytes, was an important negative biomarker for myocardial ischemia [8]. Consistent with our previous study, Sanchez-Infantes D et al reported that OSM was upregulated and adipsin was downregulated in a murine model of DM [18]. Taken together, OSM derived from adipose tissue can contribute to develop the atherosclerosis, such as calcified lesions.

Shear stress had been known to contribute to atherosclerotic plaque formation [20]. Interestingly, fluid shear stress or physical/ mechanical cues were demonstrated to activate YAP [21], [22] or the YAP–OSM pathway and induce atherosclerotic plaque formation. Therefore, the YAP–OSM pathway may promote angiogenesis or proliferation [23], [24], [25] and can play an important role in DM-related cardiovascular diseases. Based on these, the underlying mechanism of DM macroangiopathy might be the fragile microvascular proliferation in the heart through the YAP–OSM pathway. This concept needs to be examined in future studies. Furthermore, the effect can depend on the difference of the transcriptional factor which binds to YAP. In fact, we recently demonstrated the different role of the transcriptional factor which binds to YAP in the HF model of lysosomal storage disease and the HF model of DM [17], [26]. Therefore, the role of YAP might differ with each clinical condition.

Chest symptoms, such as chest pain or dyspnea, comprise the predominant manifestation of ischemic heart disease. However, accumulated evidence in recent years demonstrated that asymptomatic myocardial ischemia was relatively frequent in patients with DM [1]. Currently, there is no appropriate diagnostic method for silent myocardial ischemia in patients with DM, and this diagnostic delay often causes fatal cardiovascular events. Therefore, development of an appropriate screening method for DM-related silent myocardial ischemia is desired. Our data here suggested that 120 pg/mL of serum OSM was the potential cutoff value for screening of silent myocardial ischemia related with DM. This reliability needs to be examined in future studies.

## 5 Study limitations

Several limitations in the present study should be mentioned. First, the source of OSM remains unknown, although it is assumed to be adipose tissue. Second, because it remains unclear whether OSM is controlled only by YAP, equating serum OSM level with the activity of the YAP–OSM pathway should be done with caution and remains to be clarified. Third, our study included only the patients with normal renal function, so the relation between OSM and renal function remains unclear. These points also need to be examined in future studies.

## 6 Conclusion

Serum OSM can be a novel biomarker for CAD and may be useful for the screening of silent myocardial ischemia related with DM.

## Funding

This work was supported by Mochida Memorial Foundation for Medical and Pharmaceutical Research and the Grants-in-Aid for Scientific Research (18K15876).

## References

[1] Chiariello M., Indolfi C. (1996). Silent myocardial ischemia in patients with diabetes mellitus. Circulation.

[2] Haffner S.M., Lehto S., Rönnemaa T. (1998). Mortality from coronary heart disease in subjects with type 2 diabetes and in nondiabetic subjects with and without prior myocardial infarction. N. Engl. J. Med..

[3] Virmani R., Burke A.P., Kolodgie F. (2006). Morphological characteristics of coronary atherosclerosis in diabetes mellitus. Can. J. Cardiol..

[4] Puente A., Roffe F., Chimal J.L. (2005). Non-invasive evaluation of coronary atherosclerotic disease in patients with silent ischemia: usefulness of myocardial perfusion spect, electrical, angiographic, and imaging correlation. Arch. Cardiol. Mex..

[5] Mitevska I.P., Baneva N., Srbinovska E. (2017). Prognostic implications of myocardial perfusion imaging and coronary calcium score in a Macedonian cohort of asymptomatic patients with type 2 diabetes. Diab. Vasc. Dis. Res..

[6] Young L.H., Wackers F.J., Chyun D.A. (2009). Cardiac outcomes after screening for asymptomatic coronary artery disease in patients with type 2 diabetes: the DIAD study: a randomized controlled trial. JAMA.

[7] Muhlestein J.B., Lappé D.L., Lima J.A. (2014). Effect of screening for coronary artery disease using CT angiography on mortality and cardiac events in high-risk patients with diabetes: the FACTOR-64 randomized clinical trial. JAMA.

[8] Ohtsuki T., Satoh K., Shimizu T. (2019). Identification of adipsin as a novel prognostic biomarker in patients with coronary artery disease. J. Am. Heart Assoc..

[9] Ridker P.M., Cook N. (2004). Clinical usefulness of very high and very low levels of C-reactive protein across the full range of Framingham Risk Scores. Circulation.

[10] Wang T.J., Larson M.G., Levy D. (2004). Plasma natriuretic peptide levels and the risk of cardiovascular events and death. N. Engl. J. Med..

[11] Jones S.A., Jenkins B.J. (2018). Recent insights into targeting the IL-6 cytokine family in inflammatory diseases and cancer. Nat. Rev. Immunol..

[12] Kubin T., Poling J., Kostin S. (2011). Oncostatin M is a major mediator of cardiomyocyte dedifferentiation and remodeling. Cell Stem Cell.

[13] Zhang X., Zhu D., Wei L. (2015). OSM enhances angiogenesis and improves cardiac function after myocardial infarction. Biomed Res. Int..

[14] Zhang X., Li J., Qin J.J. (2017). Oncostatin M receptor β deficiency attenuates atherogenesis by inhibiting JAK2/STAT3 signaling in macrophages. J. Lipid Res..

[15] Kakutani Y., Shioi A., Shoji T. (2015). Oncostatin M promotes osteoblastic differentiation of human vascular smooth muscle cells through JAK3-STAT3 pathway. J. Cell. Biochem..

[16] Ikeda S., Mizushima W., Sciarretta S. (2019). Hippo deficiency leads to cardiac dysfunction accompanied by cardiomyocyte dedifferentiation during pressure overload. Circ. Res..

[17] Ikeda S., Mukai R., Mizushima W. (2019). Yes-associated protein (YAP) facilitates pressure overload-induced dysfunction in the diabetic heart. JACC Basic Transl Sci..

[18] Sanchez-Infantes D., White U.A., Elks C.M. (2014). Oncostatin m is produced in adipose tissue and is regulated in conditions of obesity and type 2 diabetes. J. Clin. Endocrinol. Metab..

[19] Wang J., Zheng Z., Huang B. (2020). Osteal tissue macrophages are involved in endplate osteosclerosis through the OSM-STAT3/YAP1 signaling axis in modic changes. J. Immunol..

[20] Cunningham K.S., Gotlieb A.I. (2005). The role of shear stress in the pathogenesis of atherosclerosis. Lab. Invest..

[21] Dupont S., Morsut L., Aragona M. (2011). Role of YAP/TAZ in mechanotransduction. Nature.

[22] Lee H.J., Diaz M.F., Price K.M. (2017). Fluid shear stress activates YAP1 to promote cancer cell motility. Nat. Commun..

[23] Zhu M., Che Q., Liao Y. (2015). Oncostatin M activates STAT3 to promote endometrial cancer invasion and angiogenesis. Oncol. Rep..

[24] Li Y., Feng J., Song S. (2020). gp130 controls cardiomyocyte proliferation and heart regeneration. Circulation.

[25] Ikeda S., Sadoshima J. (2016). Regulation of myocardial cell growth and death by the hippo pathway. Circ. J..

[26] Ikeda S., Nah J., Shirakabe A. (2021). YAP plays a crucial role in the development of cardiomyopathy in lysosomal storage diseases. J. Clin. Invest..

