# Supplementary material for: Oncostatin M is a novel biomarker for coronary artery disease – A possibility as a screening tool of silent myocardial ischemia for diabetes mellitus
Source: Int J Cardiol Heart Vasc. 2021 Jun 26;35:100829. doi: 10.1016/j.ijcha.2021.100829 (PMC8250159; doi:10.1016/j.ijcha.2021.100829)
Supplement: Supplementary data 1 [file mmc1.pdf]

Online Figure 1

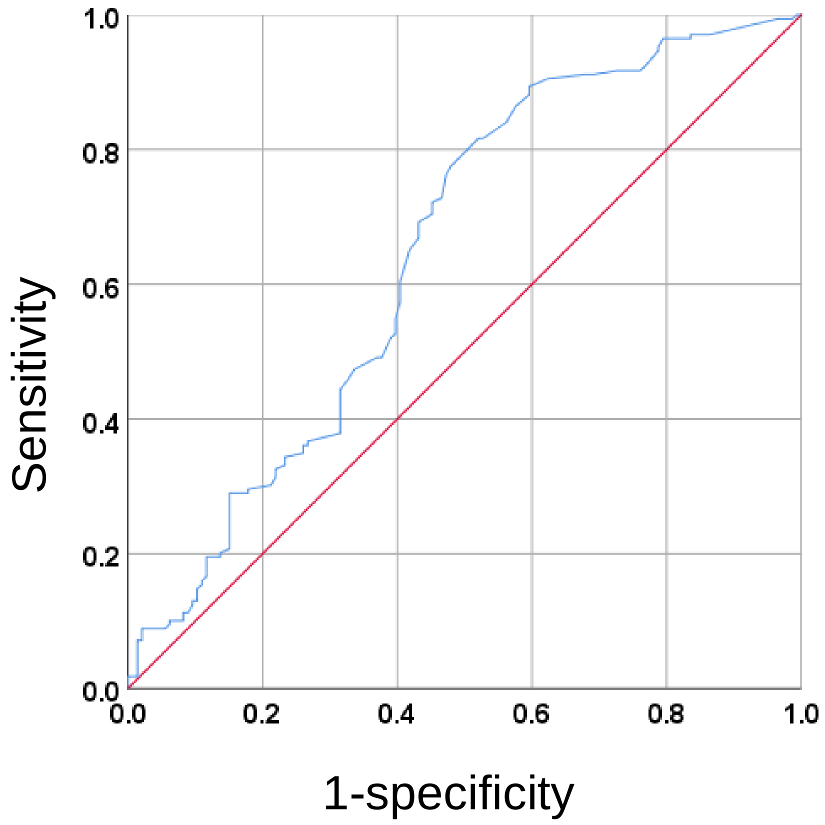

AUC：0.648（95%CI：0.586-0710）

Online Figure 1. ROC Curve of OSM for CAD

Online Figure 2

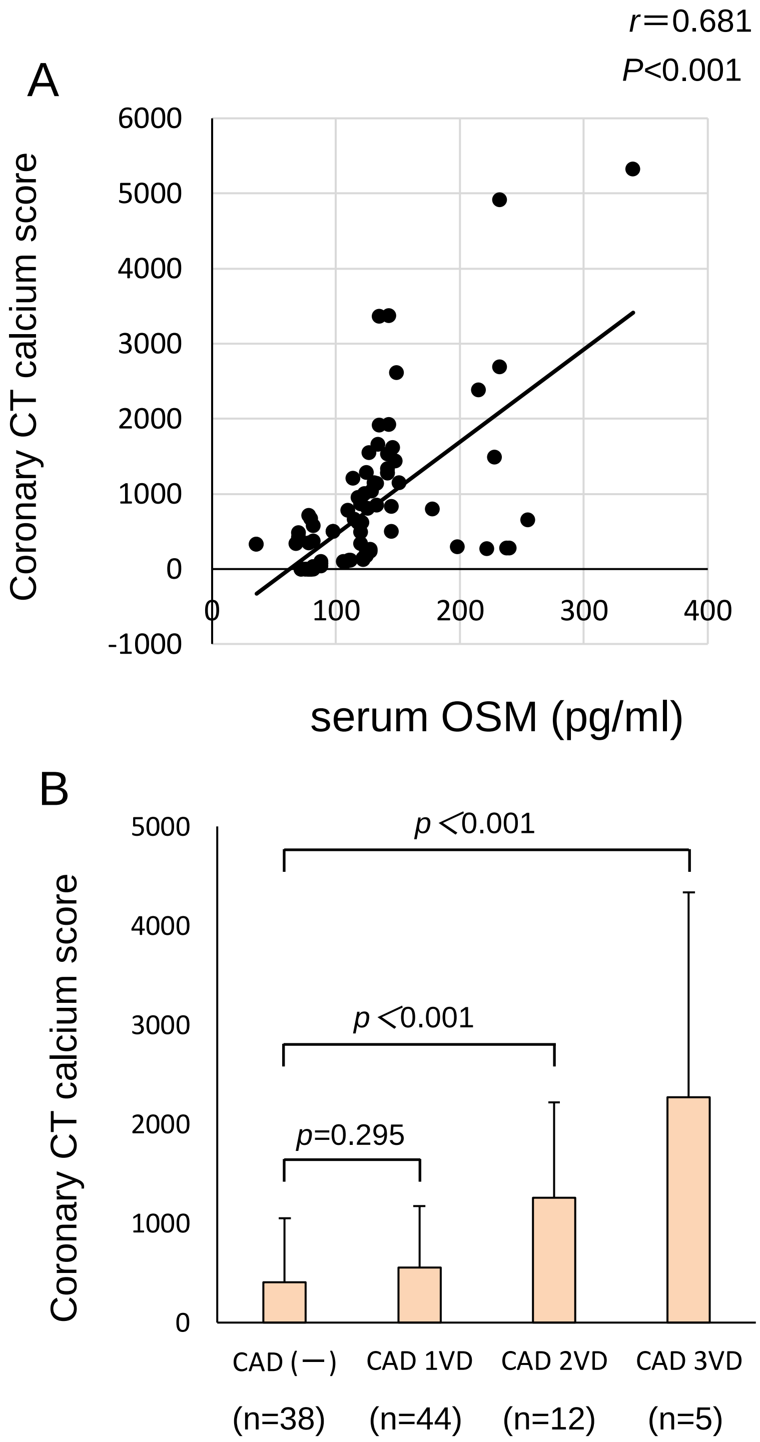

Online Figure 2. Coronary CT calcium score in the CAD patients in this study

Coronary CT calcium score was evaluated in the patients who underwent coronary CT scan before CAG. A) The significant correlation between OSM level and coronary CT calcium score. B) The association between coronary CT calcium score and CAD.

Online Figure 3

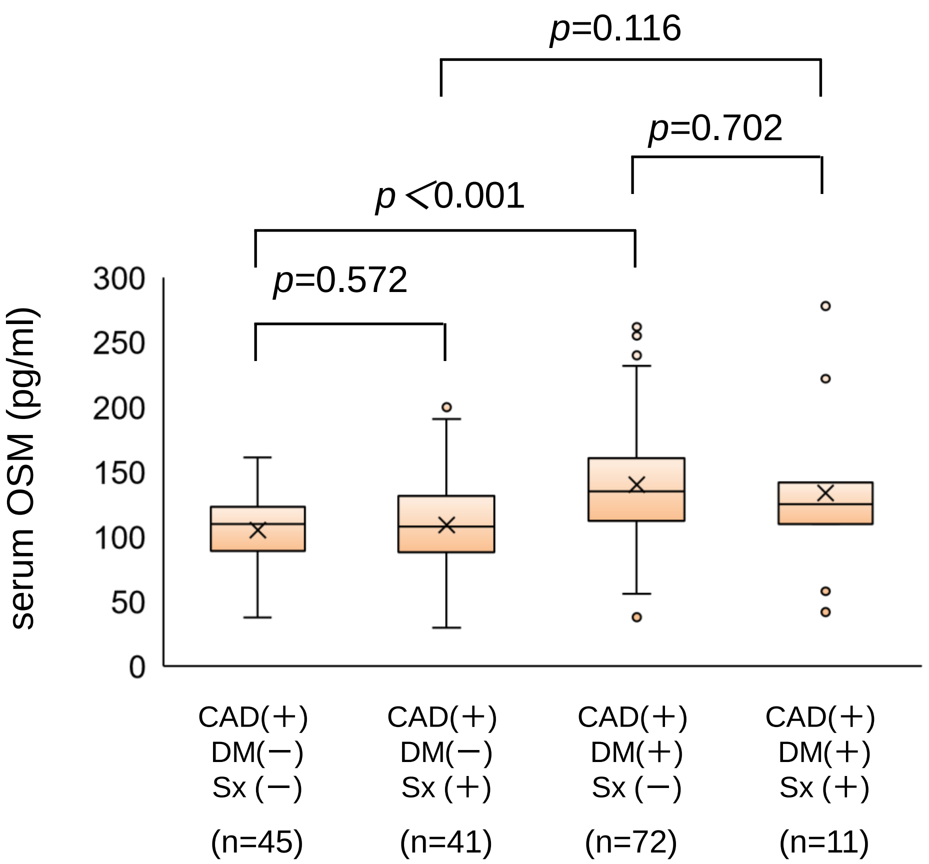

Online Figure 3. Serum OSM level in patients with DM and CAD that presented as silent myocardial ischemia

Among patients with DM and CAD, serum OSM level was significantly high in those who exhibited silent myocardial ischemia.

Online Table 1

	Male	Female	Total	
Number	246	69	315	
Age, years	71.4±9.3	77.2±5.2	72.6±8.9	<0.001
CAD (%)	127 (52)	42 (61)	169 (54)	0.174
BMI, kg/m^2	23.7±2.8	23.2±2.5	23.6±2.7	0.224
BNP, pg/ml	13.4±8.2	13.3±8.9	13.4±8.3	0.942
HbA1c, %	6.52±1.0	6.36±0.9	6.48±0.9	0.181
LVEF, %	62.3±6.6	62.4±6.6	62.3±6.6	0.761
hs-CRP, mg/dl	0.31±0.43	0.30±0.31	0.31±0.41	0.898
LDL-C, mg/dl	96.8±30.5	93.1±33.9	96.0±31.2	0.414
HDL-C, mg/dl	48.5±12.6	49.3±10.9	48.7±12.3	0.587
OSM, pg/ml	113.4±50.2	105.0±42.8	111.5±48.8	0.209
Medical history				
DM (%)	114 (46)	30 (43)	144 (46)	0.673
Hypertension (%)	215 (87)	54 (78)	269 (85)	0.058
Dyslipidemia (%)	198 (80)	50 (72)	248 (79)	0.150
Smoking (%)	125 (51)	30 (43)	155 (49)	0.281

Online Table 1 Sex differences in clinical characteristics

Online Table 2

	Univariable analysis			Multivariable analysis		
	OR	95%CI	*P*-value	OR	95%CI	*P*-value
Age	0.988	0.964-1.013	0.346			
Gender	0.686	0.398-1.182	0.175	0.587	0.332-1.038	0.067
HT	0.636	0.334-1.212	0.169			
DM	1.345	0.861-2.101	0.193			
DL	1.075	0.626-1.845	0.794			
Smoking	1.420	0.910-2.215	0.123	1.597	1.000-2.548	0.050
BMI	1.043	0.961-1.133	0.315			
BNP	0.994	0.968-1.021	0.651			
HbA1c	1.393	1.088-1.783	0.009			
LVEF	0.989	0.957-1.023	0.537			
hs CRP	0.874	0.509-1.501	0.626			
LDL	1.001	0.994-1.008	0.808			
OSM	1.012	1.006-1.017	<0.001	1.012	1.007-1.018	<0.001

Online Table 2. Factors associated with CAD（Logistic regression analysis）

Online Table 3

	Univariable analysis			Multivariable analysis		
	OR	95%CI	*P*-value	OR	95%CI	*P*-value
Age	0.991	0.959-1.025	0.618			
Gender	1.119	0.555-2.254	0.753			
HT	0.486	0.214-1.104	0.085			
DM	3.475	1.844-6.546	<0.001	0.315	0.090-1.106	0.071
DL	0.704	0.333-1.485	0.356			
Smoking	0.858	0.468-1.572	0.621			
BMI	1.089	0.979-1.210	0.116			
BNP	1.019	0.981-1.058	0.325			
HbA1c	2.796	1.897-4.121	<0.001	4.942	2.312-10.566	<0.001
LVEF	1.006	0.958-1.055	0.817			
hs CRP	0.699	0.300-1.631	0.408			
LDL	1.003	0.994-1.013	0.507			
OSM	1.017	1.008-1.025	<0.001			

Online Table 3. Factors associated with CAD-Type C lesions among CAD patients（Logistic regression analysis）
